# The Diagnostics and Management of Bronchopulmonary Sequestration: An International Survey among Specialized Caregivers

**DOI:** 10.1055/s-0044-1782237

**Published:** 2024-03-06

**Authors:** C.M. Kersten, M.D.G. Jansen, M.J.P. Zuidweg, R.M.W.H. Wijnen, T.B. Krasemann, J.M. Schnater

**Affiliations:** 1 Department of Paediatric Surgery, Erasmus MC Sophia Children's Hospital, Rotterdam, The Netherlands; 2 Medicine master's student, Leiden University Medical Centre, Leiden, The Netherlands; 3 Department of Paediatric Cardiology, Erasmus MC Sophia Children's Hospital, Rotterdam, The Netherlands

**Keywords:** congenital lung abnormalities, bronchopulmonary sequestration, surgery, surgical resection, embolization, survey

## Abstract

**Background** Our objective was to explore the treatment preferences for bronchopulmonary sequestration (BPS) among an international group of specialized caregivers.

**Methods** Sixty-three participants from 17 countries completed an online survey concerning the diagnostics, treatment, and follow-up. Recruitment took place among members of the Collaborative Neonatal Network for the first European Congenital Pulmonary Airway Malformation Trial Consortium and through the Association for European Pediatric and Congenital Cardiology working group database.

**Results** Most of the 63 participants were pediatric surgeons (52%), followed by pediatric pulmonologists (22%), and pediatric cardiologists (19%). The majority (65%) treated more than five cases per year and 52% standardly discussed treatment in a multidisciplinary team. Half of the participants (52%) based the management on the presence of symptoms, versus 32% on the intralobar or extralobar lesion localization. Centers with both surgical and interventional cardiac/radiological facilities (85%) preferred resection to embolization in symptomatic cases (62 vs. 15%). In asymptomatic cases too, resection was preferred over embolization (38 vs. 9%); 32% preferred noninterventional treatment, while 11% varied in preference. These treatment preferences were significantly different between surgeons and nonsurgeons ( *p* < 0.05). Little agreement was observed in the preferred timing of intervention as also for the duration of follow-up.

**Conclusions** This survey demonstrates a variation in management strategies of BPS, reflecting different specialist expertise. Most centers treat only a handful of cases per year and follow-up is not standardized. Therefore, management discussion within a multidisciplinary team is recommended. Recording patient data in an international registry for the comparison of management strategies and outcomes could support the development of future guidelines.

**Level of Evidence** : Level IV.

## Introduction

Congenital lung abnormalities (CLAs) include a variety of developmental disorders of the respiratory tract and pulmonary vascularization. 1 The incidence is approximately 4 in 10,000 births. A number of diagnosed cases have increased over the past decades, presumably due to the implementation of standard-of-care prenatal ultrasound imaging with better image quality. 2 3 Comprising approximately 23% of all prenatally detected CLAs, bronchopulmonary sequestration (BPS) is the second most frequent subtype. 4 A BPS consists of lung tissue that is not connected to the tracheobronchial tree and derives its blood supply from the systemic circulation instead of the pulmonary arteries. 5 6 The majority of neonates with BPS are asymptomatic at birth and the prognosis is usually good. 6 7 In case symptoms do occur, most often they consist of recurrent pneumonia, respiratory distress, or high output congestive heart failure due to right-to-left shunt. 8 9

Two separate types of BPS can be distinguished: intralobar sequestration (ILS) and extralobar sequestration (ELS). 6 An ILS shares the visceral pleura with the adjacent lobe and accounts for 75% of cases, whereas an ELS is encompassed by its own pleura, separating it from the rest of the lung and accounts for the remaining 25% of cases. 6 8 A hybrid lesion is either an ILS or an ELS which shows the characteristics of both a BPS and a congenital pulmonary airway malformation (CPAM), another type of CLA in which overgrowth of respiratory tract tissue leads to the formation of cysts. 6 10 11

BPS can be diagnosed prenatally by routine ultrasonography, when it resembles a hyper echogenic solid mass, commonly appearing like a wedge. 12 13 Doppler ultrasound is frequently used to identify the systemic feeding artery, which is pathognomonic for a BPS. 14 15 In two-thirds of cases, the lesion regresses during gestation, in some cases even becoming undetectable on consecutive ultrasonography or postnatal chest X-ray. 14 16 17 After birth, a BPS is usually confirmed by visualizing the systemic feeding artery on chest computed tomography scan (CT scan) with intravenous contrast, angiography, or magnetic resonance imaging (MRI). Occasionally, the feeding artery can be visualized on Doppler ultrasound. The optimal management of a newborn with BPS who remains asymptomatic is still under debate, whereas it is generally accepted that an intervention is indicated when symptoms occur. 5 6 7

Treatment options for symptomatic cases are surgery, endovascular embolization, or combination of both. Complete surgical resection of the lesion is considered curative and is associated with relatively low morbidity, especially in an elective setting. 5 6 7 8 18 19 20 An alternative treatment option is endovascular embolization of the systemic arterial supply to the lesion through percutaneous endovascular access, to block the blood flow to the lesion and induce necrosis and involution. 8 21 22 23 Embolization is considered a viable, less invasive alternative to surgical intervention but has not yet been properly evaluated in prospective, comparative trials. 8 Moreover, embolization can serve as treatment prior to surgical resection, by shrinking the lesion and possibly reducing the risk of intraoperative hemorrhage. 8

To date, no studies have compared the abovementioned treatment modalities and only a few follow-up studies on BPS patients are available, limiting the understanding of outcome in this patient group. 5 24 In addition, the current standard of care in BPS patients is unknown due to the absence of large cohort data and international guidelines. 25 As a first step, we conducted a survey to identify the management preferences for BPS among a European group of specialized caregivers.

## Methods

### Survey Design and Distribution

An online survey was designed in Google Forms (Google LLC, Mountain View, CA, United States), consisting of 44 questions in total, which addressed the following areas: general management, prenatal management, postnatal management, follow-up, and future research. The full survey can be found in Supplementary Materials (available in the online version). The survey was sent to all members of the Collaborative Neonatal Network for the first European CPAM Trial (CONNECT) consortium, an international collaboration of specialist caregivers aimed at improving the care for patients with congenital lung disease. 26 Moreover, the survey was dispersed in the Association for European Pediatric and Congenital Cardiology (AEPC) working group database. 27 Apart from being a member of (at least) one of these two networks, no further criteria had to be met to be able to partake in the survey.

Considering this survey did not involve the inclusion of patients or the collection of patient-related data, no ethical approval was required for this study.

### Data Management and Analysis

All responses were included for analysis. Anonymized data were stored on a secure online server and managed according to the European General Data Protection Regulation. 28 Responses to each question are shown in numbers (percentages). Analyses were performed in SPSS (version 25, IBM Corporation, Armonk, NY, United States), and significance was tested using Fisher's exact test. Significance was set at *p* < 0.05.

## Results

**Fig. 1:**
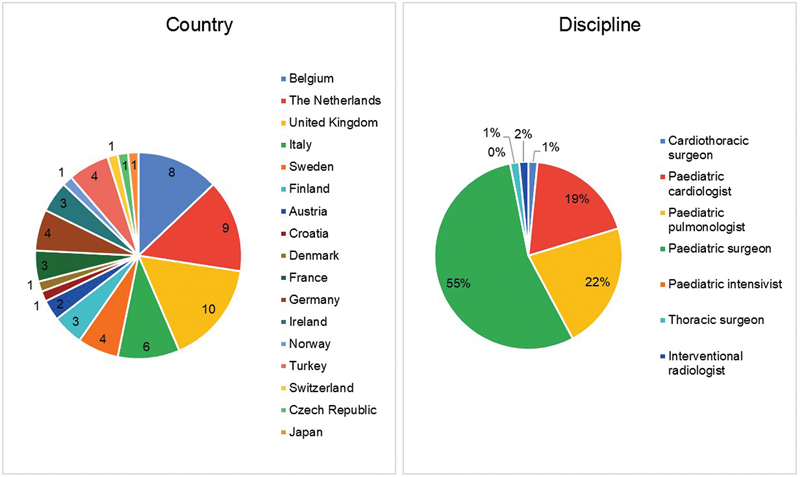
Participant characteristics.

The survey was completed by 63 participants, divided over 46 individual hospitals, from 17 countries, and consisted of 33 pediatric surgeons (52%), 14 pediatric pulmonologists (22%), and 12 pediatric cardiologists (19%), see Fig. 1 . In three cases, a maximum of three participants were associated with the same center. Most centers treated five or less BPS cases each year (65%) and performed 0 to 5 resections (79%) and between 0 and 1 embolization (67%). Pediatric surgeons and pediatric pulmonologists were available in nearly all centers (95 and 92%), followed by pediatric interventional cardiologists in 75% and interventional radiologists in 54%. There were no missing data from the survey.

### Prenatal Management

**Fig. 2:**
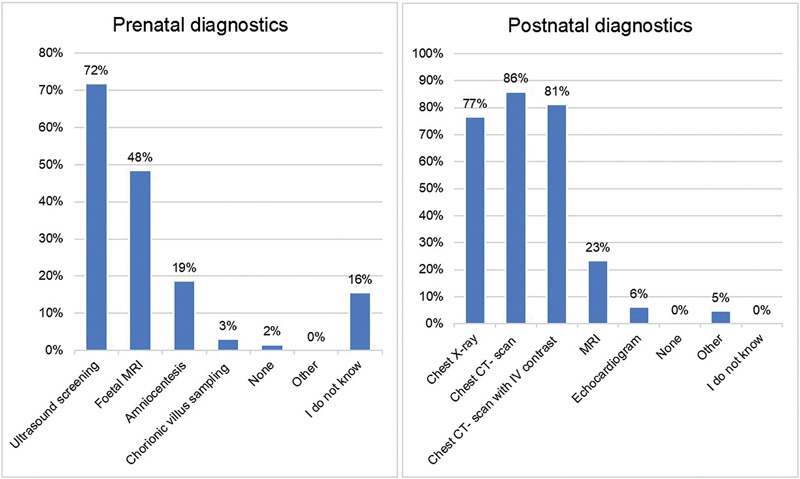
Diagnostic workup of BPS patients.

Most participants (57%) estimated that the majority of BPS cases are diagnosed prenatally, usually through ultrasonography or fetal MRI, see Fig. 2 . Parents who are expecting a child with a BPS are standardly referred to a specialized center for delivery, although up to 30% of participants stated to refer parents only occasionally or never (8%). Parental counseling is offered as a standard of care in 87%. Pregnancy termination, however, is offered only incidentally (5%). Parental preference is taken into consideration when deciding on the best treatment in 66%, and half of the participants (52%) confirmed that management strategy is always discussed in a multidisciplinary team.

### Postnatal Management

Neonates in good clinical condition without symptoms are generally observed one to several days after birth (75%), while some are discharged immediately (5%) or observed a week or longer (5%). Postnatal imaging is usually performed as the standard of care (86%) and most often consists of chest X-ray and chest CT imaging, see Fig. 2 .

**Fig. 3:**
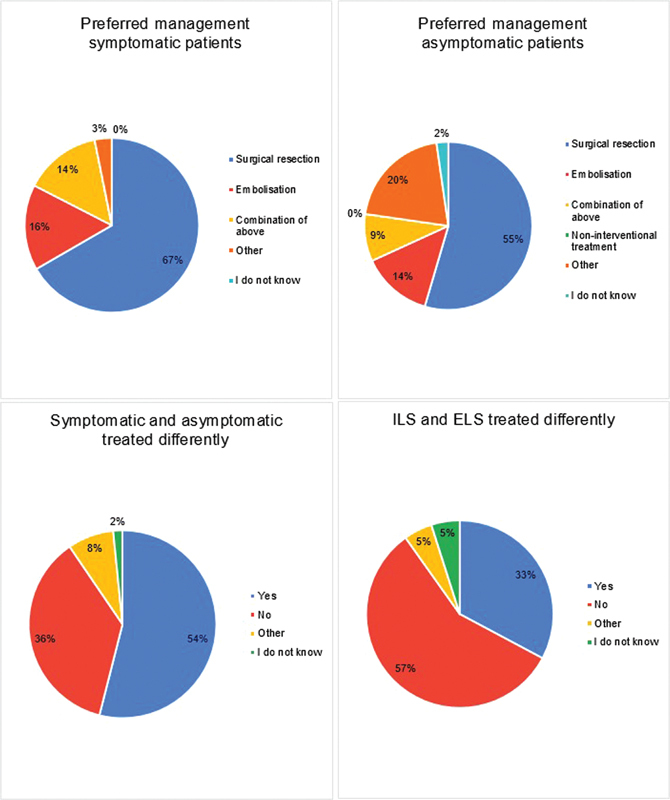
Management of BPS patients.

Half of the participants (52%) base their management strategy on the presence of symptoms, while one-third (32%) take into account the intralobar or extralobar location of the BPS. As for hybrid lesions, the majority of participants (71%) treat these like regular BPS lesions. Please see Fig. 3 for an overview of management preferences.

Most centers have access to both surgical and interventional cardiac/radiological facilities (85%). Among these centers, surgery was the preferred treatment over embolization in symptomatic cases (62 vs. 15% of participants), and a combination of surgery and embolization was chosen in 17%, see Table 1 . A significant difference was observed in these preferences between surgeons and nonsurgeons, showing a greater preference for surgery among surgeons as opposed to a greater preference for embolization or combined treatment amongst nonsurgeons ( *p* = 0.006). For asymptomatic cases, too, surgery was generally the preferred management to embolization (38 vs. 9% of participants), while 32% preferred noninterventional treatment, see Table 1 . Again, a significant difference was observed between surgeons and nonsurgeons due to a greater preference for surgery among surgeons, although the preference for noninterventional treatment was roughly comparable between these two specialist groups ( *p* = 0.04). The above-mentioned management preferences do not significantly differ between centers with both surgical and interventional facilities and centers with only surgical treatment options. Unfortunately, the sample size of the latter group was limited ( *n* = 7), see Table 2 .

**Table 1:** Management preferences among surgeons and nonsurgeons, in centers with both surgical and interventional capacities

What is the preferred management of symptomatic BPS patients?				
	Nonsurgeons	Surgeons	Total	
	*n* (%)	*n* (%)	*n* (%)	
Surgical resection	10 (40)	23 (84)	33 (62)	
Embolization	7 (28)	1 (4)	8 (15)	
Combination of above	5 (20)	4 (14)	9 (17)	
Variable	2 (8)	0	2 (4)	
Unknown	1 (4)	0	1 (2)	
Total	25	28	53	*p* = 0.006
**What is the preferred management of asymptomatic BPS patients?**				
	**Nonsurgeons**	**Surgeons**	**Total**	
	***n*** **(%)**	***n*** **(%)**	***n*** **(%)**	
Surgical resection	6 (24)	14 (50)	20 (38)	
Embolization	5 (20)	0	5 (9)	
Noninterventional treatment	7 (28)	10 (35)	17 (32)	
Variable	3 (12)	3 (11)	6 (11)	
Combination of above	3 (12)	1 (4)	4 (7)	
Unknown	1 (4)	0	1 (2)	
Total	25	28	53	*p* = 0.04

**Table 2:** Preferred treatment of centers with only surgical capacities versus centers with both surgical and interventional capacities (i.e., cardiology and/or radiology)

What is the preferred management of symptomatic BPS patients?			
	Surgery	Surgery + interventional	*p* -Value
Surgical resection	7 (100%)	33 (62%)	
Embolization	0 (0%)	8 (15%)	
Combination of above	0 (0%)	9 (17%)	
Other	0 (0%)	3 (6%)	
Total	7	53	*p* = 0.47
**What is the preferred management of asymptomatic BPS patients?**			
	**Surgery**	**Surgery + interventional**	***p*** **-Value**
Surgical resection	4 (57%)	19 (36%)	
Noninterventional treatment	1 (14%)	17 (32%)	
Other	2 (29%)	8 (15%)	
Embolization	0 (0%)	5 (9%)	
Combination of above	0 (0%)	4 (8%)	
Total	7	53	*p* = 0.64

**Fig. 4:**
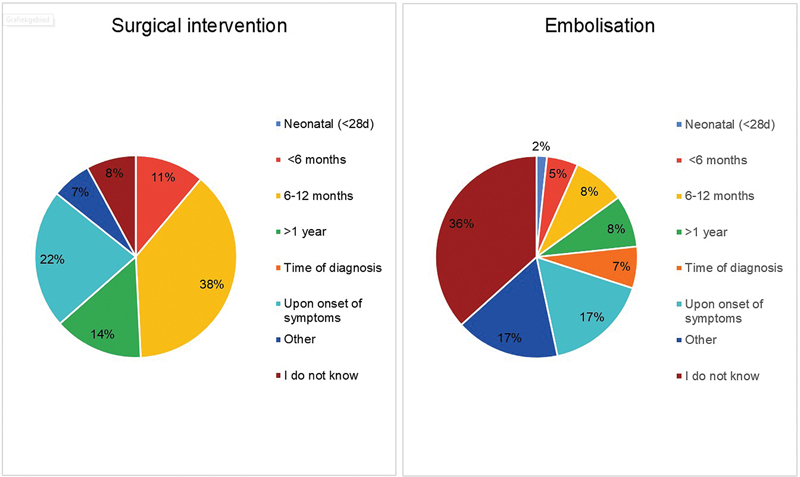
Preferred timing of intervention in BPS patients.

The timing of surgery was considered optimal either at 6 to 12 months of age (38%), upon the onset of symptoms (22%) or after the age of 1 year (14%), see Fig. 4 . Regarding embolization, more than one-third of the participants (38%) had no preference regarding the optimal timing of intervention. In addition, 37% of all participants estimated that surgical resection would be necessary after primary embolization in some cases though another 42% stated not to know this rate, see Fig. 4 .

### Follow-up

**Fig. 5:**
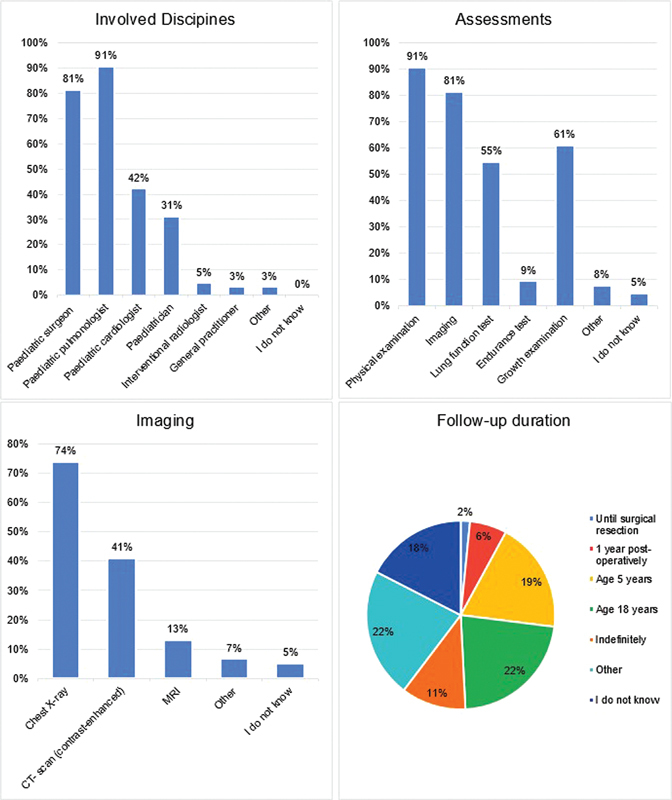
Follow-up of BPS patients.

An overview of follow-up details is shown in Fig. 5 . A structured follow-up program is offered in 75% of the centers, involving predominantly pediatric pulmonologists (89%), pediatric surgeons (78%), and pediatric cardiologists (41%). In general, the follow-up consists of physical examination (88%), imaging (80%), growth assessment (61%), and lung function tests (55%). Follow-up imaging generally includes chest X-ray (71%), CT-scan imaging (41%), and in some cases MRI (13%). The follow-up duration varies widely from one postoperative visit to monitoring up to the age of 18 years, or even indefinitely. Half of the centers (54%) apply different follow-up schemes for surgically managed children and children assigned to noninterventional treatment.

## Discussion

**Fig. 6:**
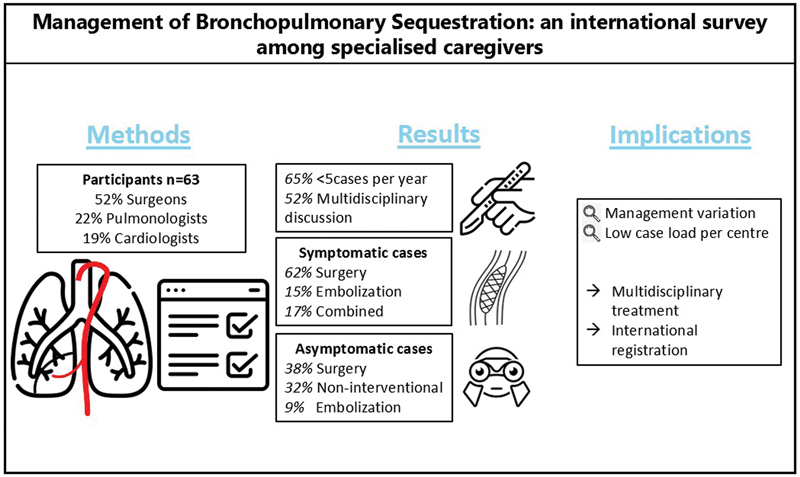
Graphical abstract.

This survey, completed by 63 participants from 17 countries, shows the general lack of standardization in Europe toward the diagnostics, management, and follow-up of children with BPS ( Fig. 6 ). A possible reason for this might be the diversity of the caregivers for this orphan disease—predominantly pediatric surgeons (52%), pediatric pulmonologists (22%), and pediatric cardiologists (19%)—combined with the different available facilities within one center. The results of this study have to be interpreted with caution but some careful conclusions may be drawn. In centers with access to both surgical and interventional treatment options, there was a preference for surgical resection, both in children with and without symptoms, as previously shown in the literature. 5 6 7 8 18 19 20 29 Regarding symptomatic children, the majority of participants considered embolization as the second best management, while opinions on the management of asymptomatic children differed—a watchful waiting approach appeared to be the main alternative to surgery for these children. Significant differences were found in the management preferences of surgical and nonsurgical participants, irrespective of the presence of symptoms. This variation in management preferences is in line with the ongoing discussion surrounding the optimal management of CLA in general, as reflected in several surveys in Europe, the United Kingdom, and Canada. 30 31 32 Consensus on the optimal management for asymptomatic children with CLA is still lacking—including children with asymptomatic BPS. 33

In general, surgical resection of BPS and other CLA is considered effective with close to zero mortality. 33 Postsurgical complications occur in 5 to 30% of cases, with risk factors being described as younger age, emergency as opposed to elective setting, and open versus thoracoscopic surgery. 20 34 35 Long-term morbidity is estimated to be low, primarily consisting of recurrent infection, symptomatic residual disease due to nonradical resection and pulmonary hyperreactivity. 20 36 Furthermore, mixed results have been reported concerning long-term functional outcomes in children who underwent surgery for CLA. 37 38 39 40

In our survey, consensus on the optimal timing of surgery was not evident: 38% of the participants deemed an age of 6 to 12 months optimal, while 22% found the onset of symptoms the ideal moment and 14% preferred to wait until the age of 1 year. Several studies support surgery before the age of 12 months because of the higher risk of perioperative complications due to possible infectious alteration in the lesion past this age. 29 41 42 43 In addition, younger age has a possible advantageous effect on postoperative compensatory lung growth, which is assumed to occur up to the age of 2 years. 44 45 46 However, lung function measurements later in the life of children who underwent early surgery do not support this assumption. 47 48 From an anesthetic view, surgery after the first year of life is often preferred, considering the elevated risk of adverse neurodevelopmental outcomes that is described following exposure to anesthesia and/or surgery during early childhood. 49 50

Embolization has several possible advantages over surgical resection. The percutaneous access is less invasive, and it does not require direct access to the thoracic cavity. This could probably result in decreased short- and long-term morbidity but data are sparse and comparative studies are lacking. 21 51 One major disadvantage of embolization is the risk of symptomatic residual disease, as shown in several small cohort studies where up to one in three children required a secondary treatment. 21 51 52 In our survey, 37% of participants estimated that surgical resection could be necessary after primary embolization but even more participants (42%) did not know the extent of this risk.

Another argument against embolization of BPS is the risk of malignant deterioration in the residual lesion. Although malignancy in the context of BPSs has not been extensively investigated, certain genes such as *KRAS* and *DICER-1* have been suggested to be associated with mucinous proliferation of lung tissue in congenital lung malformations, which in turn is considered a risk for malignancy development. 20 53 54 However, it is important to underscore that the overall risk of malignant degeneration in these lesions, although difficult to estimate, is believed to be low, and therefore, clinicians may consider not to resect all identified lesions. 55 56 57 Moreover, it is worth mentioning that the presence of malignancy in CLAs with a systemic arterial branch, like BPS and hybrid lesions, is believed to be even less frequent than in other CLAs. 58 Additional research is still needed to establish definitive associations. 59

Regarding the management of asymptomatic lesions, the risk of a lesion becoming symptomatic should be weighed against the risk of posttherapeutic complications. This risk may well differ between ILS and ELS lesions. An ILS lesion is thought to be more prone to infections since it is connected to the adjacent lung tissue by the pores of Kohn, which permit bacteria to enter the sequestration. 6 24 It has been reported that by the age of 20, 60% of ILS cases have become symptomatic with recurrent infections, and the risk of infection could therefore potentially outweigh the risk of interventional complications. 24 60 An ELS, on the other hand, is surrounded by its own visceral pleura and therefore is not directly connected to the adjacent lung tissue. An ELS will often remain asymptomatic during childhood, and some authors think it is likely to remain asymptomatic throughout life. 61 Hence, a conservative approach for ELS seems a decent option, though large-scale studies or registries are needed to confirm this. 24 60 Interestingly, in our survey, the majority (57%) of participants noted that they treat children with ILS and children with ELS in the same manner, suggesting that only few treating specialists are aware of the potential differences in natural history between the BPS subtypes. It is currently unclear with what degree of accuracy ILS and ELS can be distinguished from each other preoperatively. 8 62 63 This could explain the similar treatment of both subtypes that was observed in this survey.

To the best of our knowledge, no studies or guidelines are available concerning the best follow-up strategy of children with BPS. Nonetheless, 75% of the participants stated that a local standardized follow-up scheme is in place. Even so, clear variability was observed in the duration of follow-up, ranging from 1 year to observation up to adulthood or even indefinitely. Chest X-ray and CT-scan imaging during follow-up is standard of care in 80% of the centers, corresponding with available literature. 6 The value of regular follow-up imaging can be questioned and must be weighed against the potential harmful effects of ionizing radiation unless MRI is used as a radiation-free method. 64 65 66 In our opinion, imaging should be reserved for selected cases but is mandatory in patients that develop symptoms during follow-up. Furthermore, MRI imaging has recently been posed as a viable, radiation-free alternative. 66 67

This study has several limitations. First, the total number of participants in this survey (63) was relatively low, despite the fact that we approached specialized caregivers through two considerable international networks. Especially within the AEPC, the response rate was low at approximately 10%. The results from this survey therefore do not necessarily reflect the opinions of the complete network. Second, due to the higher response rate within the CONNECT consortium, the majority of participants were pediatric surgeons, introducing a possible bias in the results that reflects certain specialist expertise. Management preferences significantly differed between surgical participants and their nonsurgical colleagues, in accordance with earlier studies. 26 These findings stress the need for a multidisciplinary approach toward children with BPS, but this appeared to be the standard of care in only half of the participating centers in this survey. Third, the number of survey questions was limited, and the majority were multiple-choice questions, with little room for personal remarks or considerations. Through this design, we aimed to maximize the participation of clinicians and aimed to collect objective data suitable for direct analysis. Possibly this survey is a first step toward international discussions and collaboration to work toward a standardized treatment for this rare congenital abnormality.

In conclusion, this survey demonstrates a wide variation in management strategies for BPS, probably in part due to specialist expertise. The majority of centers treat five or less BPS cases per year, and there are no generally accepted follow-up standards. Discussing the management of patients with BPS in a multidisciplinary team consisting of all potentially concerned with the care of these patients should become standard. Finally, recording patient data in an international registry are desirable to gain insight into and compare management strategies and outcomes, which could probably support the development of future guidelines.

**Abbreviations**
AEPC: Association for European Pediatric and Congenital Cardiology
BPS: bronchopulmonary sequestration
CLA: congenital lung abnormality
CONNECT: Collaborative Neonatal Network for the first European CPAM Trial
CPAM: congenital pulmonary airway malformation
CT-scan: computed tomography scan
ELS: extralobar sequestration
ILS: intralobar sequestration
MRI: magnetic resonance imaging

## Notes

Highlights

- What is Currently Known about this Topic?
  Bronchopulmonary sequestration can be managed by either surgical resection, percutaneous embolization, or conservative follow-up. Current management preferences throughout Europe are currently unknown.
- What New Information is Contained in this Article?
  This multidisciplinary survey demonstrates a large variation in the management of BPS. Therefore, cases should be discussed in a multidisciplinary team and an international registry is desirable.
