# Supplementary material for: The Diagnostics and Management of Bronchopulmonary Sequestration: An International Survey among Specialized Caregivers
Source: Eur J Pediatr Surg. 2024 Mar 6;35(2):147–58. doi: 10.1055/s-0044-1782237 (PMC11932755; doi:10.1055/s-0044-1782237)
Supplement: Supplementary file 1 — Supplementary Material [file 10-1055-s-0044-1782237-s2024016869oa.pdf]

The diagnostics and management of bronchopulmonary sequestration: a European survey

Different treatment modalities are offered to patients with bronchopulmonary sequestration. With this survey we would like to evaluate which of these are applied and why specific options are chosen.

The full questionnaire takes approximately 15 minutes to complete. Would you be so kind to complete at least the first section? The second section explores the subject more in- depth. To save your time, this second section is optional.

Still, all input is equally helpful, and we would greatly appreciate receiving answers on as many questions as possible. If you cannot answer a question, please fill in 'I do not know' and continue to the next question.

If any problems should occur, please do not hesitate to contact us via connect@erasmusmc.nl.

Thank you for your participation.

Erasmus MC
SOPHIA CHILDREN'S HOSPITAL

I. General information

1. What is your email address?

____

2. What is your discipline?

- ◯ Paediatric surgeon
- ◯ Paediatric pulmonologist
- ◯ Paediatric cardiologist
- ◯ Interventional radiologist
- ◯ Other: ______________________________

In light of possible information bias, the information obtained by the next question serves only to check to what extent information comes from the same centre.

3. Where do you work (name of the centre, country, city)?

____

____

____

____

____

II. General overview

4. How many patients are treated with bronchopulmonary sequestration (BPS) in your centre each year?

- ◯ 0-1
- ◯ 0-5
- ◯ 5-10
- ◯ >10
- ◯ I do not know

 © Thieme. All rights reserved.

5. How many surgical interventions for BPS are performed each year in your centre?

- 0-1
- 0-5
- 5-10
- >10
- I do not know

6. How many percutaneous interventions (selective embolisation of systemic feeding vessel) for BPS are performed each year in your centre?

- 0-1
- 0-5
- 5-10
- >10
- I do not know

7. Is, in your centre, the management strategy of BPS patients discussed in a multidisciplinary team?

- Never
- Sometimes
- Often
- Always
- I do not know

8. Which of the following disciplines, potentially involved in the management of BPS patients, are available in your centre? (Multiple answers possible)

- [ ] Paediatric surgery
- [ ] Paediatric pulmonology
- [ ] Paediatric cardiology
- [ ] Interventional radiology
- [ ] I do not know
- [ ] Other: ____________________

III. General management

9. How many patients with a BPS present with symptoms?

- ◯ <25%
- ◯ 25- 50%
- ◯ 50- 75%
- ◯ >75%
- ◯ I do not know

10. On which types of BPS is postnatal imaging performed in your centre?

- ◯ Symptomatic cases
- ◯ Asymptomatic cases
- ◯ All cases
- ◯ I do not know
- ◯ Other: ____________________

11. What does the postnatal diagnostic workup of BPS cases include? (Multiple answers possible)

- ☐ Chest X-ray
- ☐ Chest CT- scan
- ☐ Chest CT- scan with IV contrast
- ☐ MRI
- ☐ None
- ☐ I do not know
- ☐ Other: ____________________

12. Are symptomatic and asymptomatic BPS patients treated differently in your centre?

- Yes
- No
- I do not know
- Other: ______________________

13. What is the preferred management of symptomatic BPS patients?

- Surgical resection
- Embolisation
- Combination of above
- Conservative follow-up
- I do not know
- Other: ______________________

14. What is the preferred management of asymptomatic BPS patients?

- Surgical resection
- Embolisation
- Combination of above
- Conservative follow-up
- I do not know
- Other: ______________________

15. Are intralobar sequestration and extralobar sequestration patients treated differently in your centre?

- Yes
- No
- I do not know
- Other: ______

16. Intralobar sequestration is treated by a (multiple answers possible):

- [ ] Paediatric surgeon
- [ ] Cardiothoracic surgeon
- [ ] Interventional cardiologist
- [ ] Interventional radiologist
- [ ] I do not know
- [ ] Other: ______

17. Extralobar sequestration is treated by a (multiple answers possible):

- [ ] Paediatric surgeon
- [ ] Cardiothoracic surgeon
- [ ] Interventional cardiologist
- [ ] Interventional radiologist
- [ ] I do not know
- [ ] Other: ______

 © Thieme. All rights reserved.

18. Is parental preference considered when determining the management strategy? (i.e., shared decision making)

- Never
- Sometimes
- Often
- Always
- I do not know

19. In case of (surgical) intervention, what is the preferred approach?

- Thoracoscopy
- Thoracotomy
- Embolisation
- I do not know
- Other: ______________________________

20. In case of embolisation, is a surgical resection needed afterwards?

- Never
- Sometimes
- Often
- Always
- I do not know

21. What is the child's preferred age for surgical intervention?

- Neonatal (<28d)
- <6 months
- 6-12 months
- >1 year
- Time of diagnosis
- Upon onset of symptoms
- I do not know
- Other: ______________________________

22. What is the child’s preferred age for embolisation?

- Neonatal (<28d)
- <6 months
- 6-12 months
- >1 year
- Time of diagnosis
- Upon onset of symptoms
- I do not know
- Other: ____________________

23. How often have complications occurred after surgical interventions in your centre?

- <1%
- 1-5%
- 5-10%
- >10%
- I do not know

24. What has been the most frequent complication within 30 days of surgical interventions in your centre?

- Air leak
- Infection
- Bleeding
- Effusion
- Respiratory distress
- Atelectasis
- I do not know
- Other: ____________________

25. How often have complications occurred after embolisations in your centre?

- <1%
- 1-5%
- 5-10%
- >10%
- I do not know

26. What has been the most frequent complication within 30 days of embolisations in your centre?

- Air leak
- Infection
- Bleeding
- Effusion
- Respiratory distress
- Atelectasis
- I do not know
- Other: ____________________

27. Is a standardised follow-up scheme for BPS in place at your centre?

- Yes
- No
- I do not know

© Thieme. All rights reserved.

IV. Prenatal management

28. What proportion of BPS cases in your centre is identified prenatally?

- <25%
- 25- 50%
- 50- 75%
- >75%
- I do not know

29. How is a BPS most frequently diagnosed prenatally?

- Ultrasound
- Doppler ultrasound
- Foetal MRI
- I do not know
- Other: ____________________

30. In case of prenatal diagnosis, what does further diagnostic workup include? (Multiple answers possible).

- [ ] Ultrasound screening for additional congenital anomalies
- [ ] Foetal MRI
- [ ] Amniocentesis
- [ ] Chorionic villus sampling
- [ ] None
- [ ] I do not know
- [ ] Other: ____________________

31. Are children with prenatally diagnosed BPS in your country referred to a specialised centre for delivery?

- Never
- Sometimes
- Often
- Always
- I do not know

32. Is prenatal counselling offered to parents as standard of care in your centre?

- Yes
- No
- I do not know

33. Is pregnancy termination offered in case of BPS?

- Yes
- No
- I do not know

V. Postnatal management

34. How long are asymptomatic stable neonates observed in your centre before being discharged?

- Not at all
- A day
- Several days
- A week
- More than a week
- I do not know

35. If BPS presents beyond the neonatal period, what are the main symptoms found in your centre? (Multiple answers possible)

- [ ] Recurrent pneumonia
- [ ] Fever
- [ ] Haemoptysis
- [ ] Chest pain
- [ ] Heart failure
- [ ] Incidental finding
- [ ] I do not know
- [ ] Other: ____________________

36. What proportion of patients with BPS present with an intralobar sequestration?

- <10%
- 10-25%
- 25-75%
- >75%
- I do not know

37. Do children with extralobar sequestration present with symptoms more often than children with intralobar sequestration do?

- Yes
- No
- I do not know

38. Is the management of hybrid lesions (BPS and congenital pulmonary airway malformation (CPAM)) the same as that of BPS lesions in your centre?

- Yes
- No
- I do not know

VI. Follow-up

39. Is the follow-up of patients who have undergone an intervention (e.g., surgical resection or embolisation) different from that of patients without intervention (conservative follow-up)?

- Yes
- No
- I do not know

© Thieme. All rights reserved.

40. What disciplines are involved in the follow-up of BPS in your centre? (Multiple answers possible)

- ☐ Paediatric surgeon
- ☐ Paediatric pulmonologist
- ☐ Paediatric cardiologist
- ☐ Interventional radiologist
- ☐ Paediatrician
- ☐ General practitioner
- ☐ I do not know
- ☐ Other: ______________________

41. Which of the following examinations is part of your standardised scheme for the follow-up of BPS? (Multiple answers possible)

- ☐ Physical examination
- ☐ Imaging
- ☐ Lung function test
- ☐ Endurance test
- ☐ Growth examination
- ☐ I do not know
- ☐ Other ______________________

42. If imaging is done during follow-up, what type of imaging? (Multiple answers possible)

- ☐ Chest X-ray
- ☐ CT- scan (contrast-enhanced)
- ☐ MRI
- ☐ I do not know
- ☐ Other: ______________________

43. Until what age are BPS patients generally monitored?

- Until surgical resection
- 1 year post-operatively
- Age 5 years
- Age 18 years
- Indefinitely
- I do not know
- Other: ____________________

VII. Future research

44. Are you interested in future collaboration concerning BPS projects?

- Yes, include my contact information for possible future studies
- No, please remove my contact information after this project
